# Supplementary material for: Chaining and the temporal dynamics of scientists’ publishing behaviour
Source: PLoS One. 2022 Dec 29;17(12):e0278389. doi: 10.1371/journal.pone.0278389 (PMC9799287; doi:10.1371/journal.pone.0278389)
Supplement: S1 Appendix — Details of how validation of semantic representations across fields and scientists was performed is included. (PDF) [file pone.0278389.s001.pdf]

To validate our semantic representation of scientific abstracts, we performed an analysis on the abstract embeddings at two levels: 1) the level of scientific fields, and 2) the level of individual scientists within a field. For the field level, we expect that abstracts from the same scientific field to be more similar or closer in semantic space than those across different scientific fields. In this respect, the abstract embeddings constructed from each scientific field should be discriminable or separable from those of other fields, more so than chance where chance defines a null condition that shuffles papers across the 5 fields. Similarly for the individual level, we should expect papers written by different scientists within a field to be discriminable from those by other scientists, more so than chance where chance defines a null condition that shuffles papers across scientists within each field in question.

We applied a procedure similar to the classic Fisher's discriminant analysis for the described two levels of validation. Fisher's discriminant analysis quantifies the relation between inter-class variability (or distance) and within-class variability as a ratio. A high ratio indicates high discriminability among the classes of data in question. We formalize the Fisher's discriminant in terms of a J-value to quantify discriminability among the scientific fields and among the individual scientists within a given field. J-value takes the ratio between inter-class variability and within-class variability, defined formally as follows:

$$\begin{aligned} J &= \frac{V_b}{V_w} \\ V_b &= \Sigma_c (d(\mu_c, \mu))^2 \\ V_w &= \Sigma_c \Sigma_{i \in c} (d(\mu_c, x_i(c)))^2. \end{aligned}$$

Across fields, c indicates the complete set of papers within a field (for instance, all the abstract embeddings of prominent scientists in the field of Chemistry), d denotes Euclidean distance, and μ_c represents the average embedding of all the papers within the field. μ represents the meta-mean of the average embeddings, and $x_i(c)$ refers to a specific abstract within the field. Within a single field, c represents the abstracts written by an individual scientist, μ_c is the average embedding of papers written by that scientist, μ is the meta-mean across all the scientists in the field, and $x_i(c)$ denotes the abstract embedding of a single paper written by scientist i.

We performed a permutation test where we calculated the values of J both across fields and for each individual field separately, and then shuffled papers among the fields to construct the null distribution in the first case and among the scientists (within each field) to construct the null distribution in the second case. For each permutation, we calculated the J values and compared these shuffled values to the true attested value across 1,000 shuffled trials.

We summarize the results from the shuffled validations in Figures S2 and S3. At both the levels of fields and individuals, we observed highly significant results (permutation test: $p < 0.001$) with no shuffled J values exceeding the true value. These results indicate that the abstract embeddings we constructed distinguish well both across scientific fields and among individual scientists. This is what we would expect if the abstracts accurately represent the diversity of findings across scientific fields and individuals.
