# Supplementary material for: Chaining and the temporal dynamics of scientists’ publishing behaviour
Source: PLoS One. 2022 Dec 29;17(12):e0278389. doi: 10.1371/journal.pone.0278389 (PMC9799287; doi:10.1371/journal.pone.0278389)
Supplement: S1 Table — Statistics from model evaluation against the null model in log-likelihood ratio for the award-winning scientists as described in the main text, using Mann-Whitney U test. The Mann-Whitney U test was used in place of a t-test because the data were not normally-distributed (all models outperformed the null, and this is highly significant). (PDF) [file pone.0278389.s004.pdf]

S4 Table: Summary statistics from evaluation against the null model, in prominent and randomly-sampled scientists across fields.

Population	Model	Test statistic (↓)	p-value
Physics (prominent)	kNN	3864.0	2.87×10^{-35}*
	Prototype	4116.0	1.43×10^{-33}*
	Progenitor	4956.0	1.75×10^{-28}*
	Exemplar	2100.0	1.41×10^{-47}*
	Local	5292.0	1.43×10^{-26}*
Chemistry (prominent)	kNN	1740.0	4.99×10^{-28}*
	Prototype	1680.0	2.60×10^{-28}*
	Progenitor	2760.0	5.46×10^{-19}*
	Exemplar	960.0	1.25×10^{-35}*
	Local	2640.0	6.30×10^{-20}*
Medicine (prominent)	kNN	2642.0	2.25×10^{-35}*
	Prototype	2567.0	7.44×10^{-36}*
	Progenitor	3322.0	2.59×10^{-30}*
	Exemplar	1057.0	2.10×10^{-48}*
	Local	3171.0	2.21×10^{-31}*
Economics (prominent)	kNN	740.0	1.31×10^{-16}*
	Prototype	888.0	1.69×10^{-14}*
	Progenitor	1406.0	2.40×10^{-8}*
	Exemplar	592.0	7.09×10^{-19}*
	Local	962.0	1.69×10^{-13}*
Computer Science (Prominent)	kNN	621.0	5.86×10^{-16}*
	Prototype	621.0	5.86×10^{-16}*
	Progenitor	759.0	7.96×10^{-14}*
	Exemplar	345.0	9.86×10^{-21}*
	Local	1104.0	3.15×10^{-9}*
Physics (random)	kNN	4788.0	6.48×10^{-30}*
	Prototype	5544.0	4.01×10^{-25}*
	Progenitor	5712.0	3.19×10^{-24}*
	Exemplar	3024.0	9.65×10^{-41}*
	Local	4536.0	6.75×10^{-31}*
Chemistry (random)	kNN	2700.0	1.19×10^{-19}*
	Prototype	2340.0	1.88×10^{-22}*
	Progenitor	2460.0	1.89×10^{-21}*
	Exemplar	1140.0	7.85×10^{-34}*
	Local	2580.0	1.79×10^{-20}*
Medicine (random)	kNN	3699.5	5.97×10^{-28}*
	Prototype	5964.5	8.66×10^{-15}*
	Progenitor	4605.5	4.55×10^{-22}*
	Exemplar	2944.5	4.12×10^{-33}*
	Local	4152.5	7.60×10^{-25}*
Economics (random)	kNN	925.0	4.57×10^{-14}*
	Prototype	1665.0	5.17×10^{-6}*
	Progenitor	1221.0	2.25×10^{-10}*
	Exemplar	629.0	2.16×10^{-18}*
	Local	703.0	2.99×10^{-17}*
Computer Science (Random)	kNN	931.5	1.38×10^{-11}*
	Prototype	724.5	2.01×10^{-14}*
	Progenitor	1138.5	7.16×10^{-9}*
	Exemplar	517.5	9.05×10^{-18}*
	Local	724.5	2.01×10^{-14}*
