# Supplementary material for: Chaining and the temporal dynamics of scientists’ publishing behaviour
Source: PLoS One. 2022 Dec 29;17(12):e0278389. doi: 10.1371/journal.pone.0278389 (PMC9799287; doi:10.1371/journal.pone.0278389)
Supplement: S2 Table — Tthe percentage breakdowns from the winner-take-all analyses described in the main text for the group of award-winning scientists as well as randomly-sampled scientists. The percentage of the model with the highest winning percentage across scientists within each scientific field is marked in bold. (PDF) [file pone.0278389.s005.pdf]

**S5 Table: Percentage breakdowns from winner-take-all analyses.**

| | kNN | Prototype | Progenitor | Exemplar | Local | Null |
|---|---|---|---|---|---|---|
| Physics (prominent) | 7.7% | 1.2% | 40.5% | **45.2%** | 3.0% | 2.4% |
| Chemistry (prominent) | 5.8% | 0.8% | 30.8% | **58.3%** | 1.7% | 2.5% |
| Medicine (prominent) | 2.6% | 2.0% | 22.5% | **70.9%** | 0.7% | 1.3% |
| Economics (prominent) | 17.6% | 0% | 24.3% | **51.4%** | 4.1% | 2.7% |
| Computer Science (prominent) | 37.7% | 1.4% | 10.1% | **44.9%** | 4.3% | 1.4% |
| Physics | 13.6% | 1.1% | 24.9% | **52.0%** | 1.7% | 6.8% |
| Chemistry | 8.3% | 1.1% | 29.4% | **58.3%** | 1.1% | 1.7% |
| Medicine | 7.3% | 0% | 34.0% | **50.6%** | 3.7% | 4.9% |
| Economics | 7.1% | 0.0% | 33.3% | **49.5%** | 7.1% | 3.0% |
| CS | 14.8% | 0.0% | 34.6% | **45.7%** | 0.0% | 4.9% |
