# Supplementary material for: Chaining and the temporal dynamics of scientists’ publishing behaviour
Source: PLoS One. 2022 Dec 29;17(12):e0278389. doi: 10.1371/journal.pone.0278389 (PMC9799287; doi:10.1371/journal.pone.0278389)
Supplement: Attachment — Submitted filename: Response to Reviewers.pdf [file pone.0278389.s006.pdf]

Response to the Reviews and Decision

Title: Regularity in the temporal dynamics of publishing behaviour across scientists

Manuscript Reference Number:
PONE-D-22-15379

Authors:
Emmy Liu
Yang Xu

Message from the Authors

Dear Editor,

We thank you and the reviewers for your constructive comments, which have allowed us to clarify and improve the manuscript. We have highlighted the changes to the manuscript in blue to differentiate them from the original contents.

We address each comment separately in our response. The comments we received are boxed, and our responses are written following each comment. Additionally, we also request a small change in the title of our paper to make it shorter and better reflect the central idea of chaining, which was pointed out by the reviewer. The following is the new title:

Chaining and the temporal dynamics of scientists' publishing behavior

We hope that you will find our revised manuscript satisfactory for publication at *PLOS ONE*.

Sincerely,
Emmy Liu and Yang Xu

Response To Reviewer #1

Overall Comments

> This is an interesting article highlighting the similarities in the pattern of publishing behaviour of individual scientists over time and the historical word meaning extension. To this end the authors have employed the cognitive theory chaining based on general context model.
>
> The article, though is very well written, it rather very briefly describes the computational framework. It would be better to add more analytical details which would aid the reader to better appreciate the methodology employed.
>
> The article is recommended for publication after the addition of more details concerning model formulation.

Response

We appreciate your review and recommendations on our work. We have revised the manuscript to motivate the computational framework better, and to make the formulation of models clearer.

Reviewer Comment

> It would be better to add more analytical details which would aid the reader to better appreciate the methodology employed.

Response

Thank you for your feedback, we have added further description to paragraph 1 of the "Computational Framework" section, in which we clarify our methodology and framing. We have also added additional details about SBERT, which is the embedding model we used, in the same section.

Reviewer Comment

> The article is recommended for publication after the addition of more details concerning model formulation.

Response

Thank you, we have added exposition to paragraph 5 of the "Computational Framework" section. In particular, each of the models examined is specified completely by Table 1 and Equation 1. We have made this more explicit in the manuscript in paragraph 11 of this section.

Response To Reviewer #2

Overall Comments

> The paper investigated an important 'social behaviour' using computing technologies. Basically, the topic is interesting for the academics. However, some issues should be taken into account during a major revision.
>
> 1) The motivation is not clear, most of the scientific findings are given based on one single amazing idea and the original idea cannot be predicted. So why we need this statistical research work?
>
> 2) Various methods have been used in the manuscript such as clustering method KNN, why these methods have been selected? Is the comparative study the main contribution?
>
> 3) In figure 1, yellow dot data has been marked as 2025. Where is this data from? I understand that the work is based on historical data.

Response

We would like to thank you for your feedback. We clarify our methods and motivation behind the paper: we are not seeking to predict what the amazing ideas that actually emerge will be, but rather trying to characterize the overall process that scientists use to extend their ideas. We have also added more detail to the "Computational Framework" section to make the motivation more clear to readers.

Reviewer Comment

> The motivation is not clear, most of the scientific findings are given based on one single amazing idea and the original idea cannot be predicted. So why we need this statistical research work?

Response

Thank you for this clarification question. We wish to emphasize that the purpose of the paper is not to predict what amazing single scientific ideas will arise, but rather to characterize the overall chaining process in a scientist's publications as they emerge through time. Novel scientific ideas have some relationship to the existing scientific literature and a scientist's past work, and it's this relationship between past work and future work that motivates our analysis with the temporal chaining models. This is similar to the word-sense extension models mentioned in paragraph 6 of the Introduction: we cannot predict exactly what novel sense(s) of a word will arise next, but we can characterize the process of sense extension and hence making predictions about the emerging order of senses over time. Similarly, we can characterize the overall growth of a scientist's publications over time, even if we cannot predict exactly what that scientist will do in the future.

Reviewer Comment

> Various methods have been used in the manuscript such as clustering method KNN, why these methods have been selected? Is the comparative study the main contribution?

Response

We have added exposition and motivation to the "Computational Framework" section of the manuscript. These methods were selected based on past work in word-sense extension, and comparative study is the main contribution, as all of these extension mechanisms are cognitively plausible and based on psychological research. Another contribution is that we find the best model to be the exemplar model, which mirrors the findings in word-sense extension, and suggests that this may be a natural and general way in which people extend concepts.

Reviewer Summary

> In figure 1, yellow dot data has been marked as 2025. Where is this data from? I understand that the work is based on historical data.

Response

We have data up until 2020, and the plot has now been fixed, since it was caused by matplotlib's automatic label generation.
